# Use of oral GnRH antagonists combined therapy in the management of symptomatic uterine fibroids

**DOI:** 10.52054/FVVO.15.1.059

**Published:** 2023-03-31

**Authors:** A Di Spiezio Sardo, F Ciccarone, L Muzii, G Scambia, M Vignali

**Affiliations:** Department of Public Health, School of Medicine, University of Naples Federico II, Via Pansini 5, Naples, Italy; Department of Woman, Child and Public Health, Fondazione Policlinico Universitario A. Gemelli IRCCS, Largo Agostino Gemelli 8, 00168 Rome, Italy; Department of Mother, Child and Urological Sciences, “Sapienza” University of Rome, Viale del Policlinico 155, 00161 Rome, Italy; Department of Biomedical Sciences for Health, University of Milan, Macedonio Melloni Hospital, Via Macedonio Melloni 52, 20129, Milan, Italy

**Keywords:** Uterine fibroids, GnRH antagonists, epidemiology, Relugolix

## Abstract

Uterine fibroids have an impact on women’s lives due to their high prevalence, physical symptoms, their consequences on patients’ emotional and psychological well-being and loss of work productivity.

The choice of therapeutical approaches varies depending on several factors, and therefore should be applied individually. Currently, there is an unmet need for good, reliable, uterine-sparing options.

The oral GnRH antagonists (Elagolix, Relugolix, Linzagolix) represent a new alternative for the medical management of hormone-dependent gynaecological diseases such as uterine fibroids or endometriosis. They rapidly bind to the GnRH receptor, block endogenous GnRH activity and directly suppress LH and FSH production, avoiding unwanted flare-up effects.

Some GnRH antagonists are marketed in combination with hormone replacement therapy add-back to counteract hypo-oestrogenic side effects. According to the registration trials, once-daily GhRH antagonist combination therapy results in a significant reduction in menstrual bleeding, as compared with placebo, and preserves bone mineral density, for up to 104 weeks. Further studies in the long term are needed to evaluate the whole impact of medical treatment of uterine fibroids on the management of this common women’s disease.

## Introduction

Uterine fibroids (UF) are one of the most important problems in gynaecological practice in terms of prevalence and burden and are the main cause of hysterectomy (Wise and Laughlin-Tommaso, 2016). In women by the age of 50, it is believed that their prevalence is approximately 70 to 80% (Baird et al., 2003). In Western Europe, their prevalence ranges from 11.7% to 23.6% (Downes et al., 2010). The most common symptoms related to UF include abnormal uterine bleeding with the associated risk of anaemia. In particular, one-third of women with UF suffer from heavy menstrual bleeding (HMB) (Al-Hendy et al., 2017), infertility and other pregnancy-related complications. Other common complaints include pelvic pain, urinary and intestinal pressure symptoms (Buttram and Reiter, 1981). The chronic nature of UF symptoms can ultimately severely impact patients’ emotional and psychological well-being, self-image and self-worth (Ghant et al., 2015). Moreover, many women report fatigue, missing work hours and loss of productivity because of UF (Borah et al., 2013, Hasselrot et al., 2018).

The choice of the most suitable therapeutic approach for UF, be it surgical, radiological (as High-Intensity Focused Ultrasound, Magnetic Resonance Guided - Focused Ultrasound Surgery, Uterine artery embolisation) or medical, starts with the interaction between the doctor and the patient. The clinician’s recommendation must consider disease-specific and patient-specific factors to establish the best risk/benefit balance for that subject (Fernandez, 2018). The cost-effectiveness of each therapy is also an aspect of considerable importance (Miller, 2009; Malone, 1969) since in the EU the cost of a hysterectomy was estimated at 5.500 €/procedure (Fernandez et al., 2009). In Italy, approximately 66% of operations for UF were hysterectomies (Di Carlo et al., 2016).

## Current management and unmet needs

Life can only be understood backwards, but it must be lived forwards (S. Kierkegaard)

National guidelines highlight the role of the physician who analyses, based on his/her experience and knowledge, the best balance between the medical and the surgical approaches, and their risks and benefits. Consideration is given not only to the costs/benefits and advantages/ disadvantages in the short term, but also and more importantly, in the medium and long term, especially when considering the morbidity and mortality rates for each surgical procedure and, to a lesser extent, medical treatment. The criteria vary in relation to several different factors, and thus the different approaches must be applied to the individual clinical case, in a perspective scenario (Conoscenti et al., 2017; Di Spiezio et al., 2019). Currently available medical therapies, radiological interventions and surgical strategies are described in Table I.

**Table I:** Medical, surgical and alternatives strategies for UF management.

Medical strategies	Surgical strategies	Alternatives to surgery
For symptomatic bleeding and pain with marginal effects on fibroid growth Tranexamic acid Oral contraceptives	Myomectomy (laparoscopic, hysteroscopic, abdominal, vaginal or robotic)	Uterine artery embolization
Hormonal treatments with proved effect on bleeding control and fibroid reduction Cabergoline SERMs	Hysterectomy (laparoscopic, vaginal, abdominal or robotic)	MR guided High Intensity Focused ultrasound
Hormonal treatments with greatest effects on volume and bleeding symptoms SPRMs GnRH agonists		

With regards to non-medical strategies, although conservative methods of treatment to preserve the uterus and fertility are often preferred, the likelihood of re-intervention is a recognised challenge with these approaches, with the added burden of cost, risk and compromised emotional wellness of the patient.

GnRH agonists were one of the first medical therapies to be used in the treatment of fibroids. They are still available but there are some issues to be considered, such as the initial flare-up effect, which causes an unfavourable rise in gonadotrophins and subsequently in sex steroids in circulation. Clinically, the flare effect can initially cause worsening of the bleeding profile. Another issue is the rebound effect that will appear with treatment discontinuation. However, the most significant limitation of long-term use of GnRH treatment is the profound hypo-oestrogenic side effects which when prolonged, can lead to bone loss (Farris et al., 2019).

The development of new medical treatments, such as the selective progesterone receptor modulator (SPRM) ulipristal acetate (UPA), allowed a reduction or postponement of surgeries.

Trend analysis suggests that surgery is becoming slightly less common among women with newly diagnosed fibroids; 34.8% of women diagnosed in 2011 were treated surgically, against only 26.0% of women diagnosed in 2015 (Bonafede et al., 2018). Although many patients have been successfully treated with UPA, even in the long term, achieving good control of UF-related symptoms, there have been concerns about severe liver toxicity. Hence, the use of UPA is now very restricted. In an Italian study, while the proportion of surgeries performed for uterine leiomyomas before the UPA suspension was 7.5% (4.5% of minor surgeries and 11.2% of major surgeries) it rose to 15.4% after the UPA suspension (15.2% of minor surgeries and 15.6% of major surgeries) (Indraccolo et al., 2019).

Therefore, there is currently an unmet need for effective and safe uterine-sparing options. In fact, among women between 40-49 years, nearly half of the patients are no longer willing to accept the trauma of a total hysterectomy for the management of a benign condition and 81% are concerned about surgery (Borah et al., 2013). But there is also an unmet need in terms of economic impact. The incorporation of SPRMs has become cost-effective for the healthcare system, as various cost-analysis studies assert (Geale et al, 2017). A simulation study in the Italian National Health System (NHS), showed that intermittent therapy with UPA was the dominant therapeutic option compared to surgery. In the base case (4 treatment cycles, over 1 year and 8 months), there was an estimated saving of 896€ per patient and an improvement in the quality of life with an overall increase of 0.006 QALYs per patient suffering from UF with moderate to severe symptoms (Colombo et al., 2019).

## New approaches

Every new beginning comes from some other beginning’s end (Seneca)

OThe oral GnRH antagonists (Elagolix, Relugolix, Linzagolix) represent a new alternative for the medical management of hormone- related gynaecological diseases such as UF and endometriosis. The efficacy is based on the fast and dose-related inhibition of circulating oestrogen levels, without the initial increase observed with GnRH agonists. GnRH antagonists rapidly bind to the GnRH receptor, block endogenous GnRH activity and directly suppress LH and FSH production, avoiding unwanted flare-up side effects (van Poppel and Klotz, 2012).

Moreover, as with GnRH agonists, the concurrent provision of oestrogen/progestin replacement therapy, or add-back therapy (ABT) to these compounds is needed to prevent the side effects of hypo-oestrogenism (mainly bone loss, hot flushes), and to allow long-term use.

Similar phase 3 clinical trials have been developed for Elagolix (Elaris Trials), Linzagolix (Primrose trials), and Relugolix (Liberty trials). All these trials share the same primary endpoint: UF symptom control. All three compounds have demonstrated excellent control of fibroid-related heavy menstrual bleeding (HMB). The three drugs have different half-lives: Elagolix (4-6h), Linzagolix (15-18h), Relugolix (25 to 65h). Elagolix Combination Therapy (300mg Elagolix + 1mg estradiol + 0.5mg norethindrone acetate / 300mg Elagolix) has been approved by the FDA for the management of heavy menstrual bleeding associated with uterine leiomyomas in premenopausal women. It is not yet available in Europe.

Linzagolix 100mg or 200mg has been very recently approved by EMA and is indicated for the treatment of moderate to severe symptoms of uterine fibroids in adult women of reproductive age (SMPC). It is recommended to be combined with a concomitant hormonal add-back therapy (1mg estradiol + 0.5mg norethisterone acetate tablet once daily) at 100mg or, if needed, 200mg once daily. For women in whom ABT is not recommended or who prefer to avoid hormonal therapy, 100mg once daily or 200mg once daily can be used but only for short-term use (< 6 months) due to the risk of bone mineral density (BMD) decrease with prolonged use (SMPC).

Two identical phase 3 trials were conducted to confirm the efficacy and safety of Linzagolix at full-suppression (200mg) and partial-suppression (100mg) doses with or without hormonal add-back therapy compared with placebo for the treatment of symptomatic uterine fibroids (Stewart et al., 2020). Linzagolix (100mg or 200mg) with or without add- back therapy significantly reduced HMB, the most common adverse events up to 24 weeks were hot flushes (Donnez et al., 2022).

Relugolix Combination Therapy (Relugolix CT) contains 40mg Relugolix + 1mg estradiol (as hemihydrate) + 0.5mg norethisterone acetate. It is licenced for the treatment of moderate to severe symptoms of uterine fibroids in adult women of reproductive age (SMPC). The dosage is one tablet daily.

The advantages of GnRH antagonist combination therapy are oral route of administration (easier than subcutaneous), avoidance of the initial flare of GnRH agonists and prevention of hypo- oestrogenic side effects due to a very low hormonal dosage ABT. The “oestrogen threshold” (Friedman et al., 1990) allows an optimal risk/benefit balance; an oestrogen concentration sufficient to limit the effects of hypo-oestrogenism due to the antagonist alone (hot flushes, detrimental effect on bone mass), but limited enough to avoid fibroid growth with the subsequent symptomatology.

The phase 3 double-blind trials (LIBERTY 1 & LIBERTY 2) demonstrated a higher proportion of women with UF and HMB responding with <80mL menstrual blood loss (MBL)/cycle and at least a 50% reduction in the Relugolix CT group. In addition, significantly more women achieved amenorrhoea, improved haemoglobin levels when presenting with anaemia at baseline, significantly reduced pain associated with UF and improved patient-reported symptom severity scale scores. The difference between Relugolix CT and placebo in reducing uterine volume was statistically significant (Al-Hendy et al., 2021). A very recent publication showed that Relugolix CT had clinically meaningful effects on women’s experience of uterine leiomyoma–associated pain (Stewart et al., 2022).

After at least one month of use, Relugolix CT inhibits ovulation in women taking the recommended dose and provides adequate contraception (SMPC).

The incidence of adverse events with Relugolix CT was similar to that observed with the placebo but most significantly, Relugolix CT maintained bone mineral density (BMD) over 24 weeks (Al- Hendy et al., 2021). The results of the extension study at week 52 demonstrated that among women treated with Relugolix CT, 87.7% were responders over the last 35 days of treatment, with a 90% reduction in MBL volume at week 52. There were no safety signals reported for Relugolix CT and lumbar spine BMD was maintained over 52 weeks of treatment (Al-Hendy et al., 2022). Longer-term data will be published shortly. According to the Summary of Product Characteristics, BMD was preserved in the lumbar spine of women treated with Relugolix CT up to 104 weeks of treatment (SMPC).

In conclusion, it can be affirmed that GnRH antagonist combination therapy represents a potential long-term treatment for women with HMB associated with UF; the combination therapy may achieve favourable control of symptoms while reducing secondary effects. Lastly, the opportunity to associate, the GnRH antagonist and the ABT in a single daily tablet is an important requirement for long-term adherence to treatment.

Which women are candidates for GnRH antagonist combination therapy? Therapeutical decisional algorithms will be developed in time, but currently, we can affirm that in symptomatic women, clinicians should consider first trying medical treatment before surgery. In particular, we can identify two main categories of symptomatic women who could benefit from this medical therapy: i) perimenopausal women and ii) women of reproductive age, irrespective of their fertility desire, trying to ameliorate their uterine and health conditions. Furthermore, those eligible for treatment with this combination therapy are women with absolute or relative contraindications to surgery or women who decline or need to delay surgery (“bridging” medical therapy). Over time, further potential treatment options will become available.

## Conclusions

It is not the ship so much as the skilful sailing that assures the prosperous voyage (G.W. Curtis)

The modern approach to UF is shifting from a lesion-oriented vision to a patient-oriented vision. This means that healthcare providers need to consider the impact of UF and treatments on women’s lives: women pursuing a career, responsible for their own household, often serving as caregivers for their parents and children, aiming for better sexual health and looking for pregnancy. The current approach must take into account women’s problems, their needs, fears and expectations and as such a holistic approach with medical treatment should always be considered as the first-line treatment.

The unmet need created by the reduced UPA indications, with the consequent increase of potentially avoidable surgeries, can be approached differently today with a reassuring patient- tailored, combined medical therapy, able to control moderate/severe symptoms and at the same time avoid secondary effects. Available clinical data on new GnRH antagonists combined therapy supports their use in these new circumstances. However, further reseach is needed to evaluate the full impact of these new medical treatments on uterine fibroids.
